# *Syzygium campanulatum* korth methanolic extract inhibits angiogenesis and tumor growth in nude mice

**DOI:** 10.1186/1472-6882-13-168

**Published:** 2013-07-11

**Authors:** Abdalrahim FA Aisha, Zhari Ismail, Khalid M Abu-Salah, Jamshed M Siddiqui, Gheniya Ghafar, Amin Malik Shah Abdul Majid

**Affiliations:** 1 Department of Pharmaceutical Chemistry, School of Pharmaceutical Sciences, Universiti Sains Malaysia, Minden 11800, Pulau Pinang, Malaysia; 2 Department of Pharmacology, School of Pharmaceutical Sciences, Universiti Sains Malaysia, Minden 11800, Pulau Pinang, Malaysia; 3 King Abdulla Institute for Nanotechnology, King Saud University, Riyadh 11451, Saudi Arabia; 4 Department of Pharmaceutical Chemistry, International Islamic University Malaysia, 25200 Kuantan, Pahang, Malaysia; 5 Australian Institute for Nanotechnology and Bioengineering, University of Queensland, Queensland 4072, Australia

## Abstract

**Background:**

*Syzygium campanulatum* Korth (Myrtaceae) is an evergreen shrub rich in phenolics, flavonoid antioxidants, and betulinic acid. This study sought to investigate antiangiogenic and anti-colon cancer effects of S.C. standardized methanolic extract.

**Methods:**

Betulinic acid was isolated from methanolic extract by crystallization and chromatography techniques. S.C. methanolic extract was analyzed by UV-Vis spectrophotometry, FTIR, LC-MS, and HPLC. Antiangiogenic effect was studied on rat aortic rings, matrigel tube formation, cell proliferation and migration, and expression of vascular endothelial growth factor (VEGF). Antitumor effect was studied using a subcutaneous tumor model of HCT 116 colorectal carcinoma cells established in nude mice.

**Results:**

Analysis by HPLC, LC-MS and FTIR confirm presence of betulinic acid in S.C. methanolic extract. Quantitative analysis by HPLC indicates presence of betulinic acid in S.C. extract at 5.42 ± 0.09% (w/w). Antiangiogenesis study showed potent inhibition of microvessels outgrowth in rat aortic rings, and studies on normal and cancer cells did not show any significant cytotoxic effect. Antiangiogenic effect was further confirmed by inhibition of tube formation on matrigel matrix that involves human endothelial cells (IC_50_ = 17.6 ± 2.9 μg/ml). S.C. extract also inhibited migration of endothelial cells and suppressed expression of VEGF. *In vivo* antiangiogenic study showed inhibition of new blood vessels in chicken embryo chorioallantoic membrane (CAM), and *in vivo* antitumor study showed significant inhibition of tumor growth due to reduction of intratumor blood vessels and induction of cell death.

**Conclusion:**

Collectively, our results indicate *S. campanulatum* as antiangiogenic and antitumor candidate, and a new source of betulinic acid.

## Background

**Figure 1:**
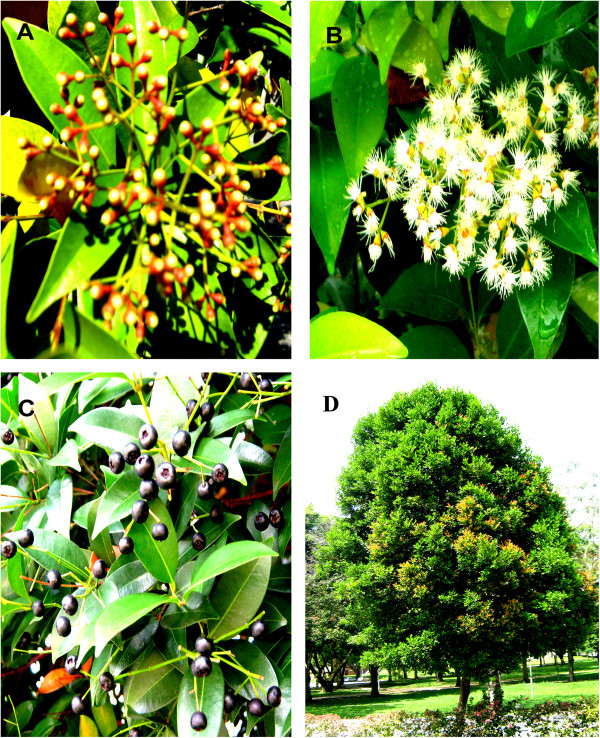
**Different parts of *Syzygium campanulatum *Korth.** Fruit buds **(A)**, flowers **(B)**, ripe fruits **(C)**, and whole tree **(D)**.

*Syzygium campanulatum* Korth (S.C.) is an evergreen shrub from the family Myrtaceae. It is known as “kelat paya” in Malaysia and Singapore where it is frequently grown as a hedge. The shrub is adapted to rapid growth under harsh conditions and can grow into tree when left alone (Figure 1) [1]. The fruits look like black berries, and can be seen from April-May and December-January. S.C. exists in 2 varieties that can be distinguished by the color of the young leaves and flowers; the first variety has yellow leaves and white-creamy flowers, and the second variety has red leaves and red flowers.

Angiogenesis is a critical process in various physiological conditions such as wound healing, female reproductive system, and embryonic development. It also plays an important role in various pathological conditions including growth and metastasis of solid tumors, rheumatoid arthritis, proliferative diabetic retinopathy, and psoriasis [2,3]. Inhibition of angiogenesis, which was suggested by Judah Folkman in 1971 [4], is now considered one of the most promising strategies to combat cancer. Recently, there has been a great interest in angiogenesis modulators for therapy of several angiogenesis related disorders. In this context, several plant-derived compounds have shown promising antiangiogenic and antitumor effects such as ursolic acid [5], oleanolic acid [6], lupeol [7], betulinic acid [8], green tea catechins [9], resveratrol from grapes [10], quercetin [11], rosmarinic acid [12], genistein [13] and curcumin [14].

**Figure 2:**
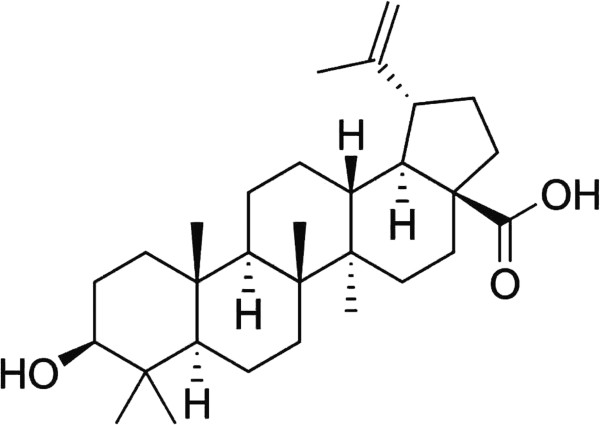
Chemical structure of betulinic acid.

Despite the widespread availability of S.C. in Malaysia, Singapore and neighboring countries, there is scarcity of data about its medicinal use and pharmacological effect. In a previous screening study, S.C. extracts exhibited potent inhibition of microvessels outgrowth in rat aortic rings which indicates possible antiangiogenic effect [15]. Therefore, this study was undertaken in order to standardize the S.C. methanolic extract including isolation of 3β-3-Hydroxy-lup-20(29)-en-28-oic acid (betulinic acid, BA) (Figure 2), determination of total phenolics, total flavonoids, BA content, and UV-Vis and FTIR fingerprints of the leaf extract. The study also sought to investigate the mechanism of angiogenesis inhibition, and anti-colon cancer effect of S.C. standardized extract. To our knowledge, this is the first study that addresses standardization and the pharmacological activity of *S. campanulatum.*

## Methods

### Cell lines and reagents

HCT 116 human colorectal carcinoma, MCF-7 human breast cancer, CCD-18Co human normal fibroblasts, and MCF-10A human normal epithelial cell lines were purchased from American Type Culture Collection (ATCC; Manassas, Virginia). Human umbilical vein endothelial cells (HUVECs) and endothelial cell medium (ECM) supplied with endothelial cell growth supplements (ECGS) were obtained from ScienCell (Carlsbad, California). RPMI 1640, DMEM, MEM and M199 cell culture media, fetal bovine serum (FBS), MEM vitamins, and non-essential amino acids were obtained from Bio-Diagnostics (Petaling Jaya, Selangor, Malaysia). Human VEGF assay kit was obtained from IBL (Aramachi, Takasaki-Shi, Gunma, Japan). Betulinic acid, ursolic acid (UA), oleanolic acid (OA), penicillin/streptomycin (PS) solution, XTT reagent with phenazine methosulfate (PMS), suramin, amphotericin B, aprotinin, 6-aminocaproic acid, L-glutamine, thrombin, human insulin, epidermal growth factor, and gentamicin were obtained from Sigma–Aldrich (Kuala Lumpur, Malaysia). Fibrinogen was obtained from Calbiochem (Shah Alam, Selangor, Malaysia). Matrigel matrix (10 mg/ml) was obtained from SABiosciences (Frederick, Maryland). Analytical and HPLC grade solvents were obtained from Avantor Performance Materials (Petaling Jaya, Selangor, Malaysia).

### Plant material and extraction

S.C. leaves were collected from the main campus of Universiti Sains Malaysia (USM) during November 2008. The plant was authenticated by the Herbarium of School of Biological Sciences, USM, where a voucher specimen was deposited (Ref. No. 11047). Leaves were washed, oven-dried at 40°C for 4 days, and grinded into fine powder. The powder (550 g) was macerated in 2L methanol for 48 h in water bath at 50°C with intermittent shaking. The extract was filtered and methanol was evaporated at 50°C using rotavapor, and further dried at 50°C for 48 h.

### Cell culture

HUVECs were cultured in ECM containing 5% FBS, 1% PS and 1% ECGS; HCT 116 cells were cultured in RPMI 1640 containing 10% FBS and 1% PS; MCF-7 and CCD-18Co cells were cultured in DMEM supplemented with 10% FBS and 1% PS. MCF-10A cells were cultured in MEM enriched with 10% FBS, 1% PS, 1% sodium pyruvate, 1% nonessential amino acids, 1% L-glutamine, 1% MEM vitamins, human insulin (5 μg/ml), and epidermal growth factor (EGF) (100 ng/ml). Cells were propagated as monolayer at 37°C and 5% CO_2._

### Phytochemical analysis

#### Estimation of total phenolics, total flavonoids and antioxidant effect

Total phenolics content was estimated using a colorimetric assay [16]. Extract (100 μl at 1.0 mg/ml in methanol) was added to 750 μl Folin-Ciocalteau reagent and incubated for 5.0 min in the dark at RT. Subsequently, 750 μl sodium bicarbonate (60 g/L) was added, incubated in the dark at 30°C for 90 min, and absorbance was measured at 725 nm. Gallic acid was used as a standard (50–1600 μg/ml), and the results are expressed as %w/w gallic acid equivalents.

Total flavonoids content was determined using quercetin as a standard [17]. The standard and extracts (500 μl) were added to 0.1 ml 10% (w/v) of aluminium chloride, 0.1 ml of 1.0 M potassium acetate, 1.5 ml methanol and 2.8 ml water. Potassium acetate and aluminium chloride were replaced with water in the blank reaction. The reaction mixture was incubated for 30 min at RT, and absorbance was taken at 415 nm. The results are presented as %w/w of total flavonoids.

DPPH scavenging activity was determined as described previously [18]. DPPH at a final concentration of 50 μM was added to S.C. extract at 12.5–200 μg/ml, incubated in the dark at 30°C for 30 min. Subsequently, absorbance was measured at 517 nm, and DPPH scavenging effect was calculated as the following;

$$\begin{array}{l} \text{DPPH}\;\text{scaveniging}\;\text{effect}\;\\ \;=\;(1-(\text{absorbance}\;\text{of}\;\text{samples}\;-\text{blank})/\\ \;(\text{absorbance}\;\text{of}\;\text{negative}\;\text{control}-\text{blank}))\times 100. \end{array}$$

The results are presented as mean percentage inhibition ± SD (n = 3).

#### Isolation and characterization of betulinic acid

BA was isolated from S.C. methanolic extract as described previously with some modifications [19]. In brief, 50 g of the methanolic extract was dissolved in 150 ml methanol and was kept at 2–8°C for overnight. Subsequently, the resulted solid was collected by filtration, and the filtrate was concentrated again and kept at 2–8°C for overnight. The solid from both cycles was pooled, and washed 3× with ice-cold methanol to give 2.5 g of BA-rich fraction. BA was further purified from this fraction by column chromatography as the following; 1 g of the fraction was dissolved in 10 ml of 1:1 methanol: chloroform and mixed with 5 g silica gel 60 (0.063–0.200 mm), the solvent was evaporated in a fume hood and the mixture was further dried for 30 min at 50°C. The column (20” × 1”) was packed with 50 g silica gel 60 (0.063–0.200 mm) prepared in the mobile phase. Elution was performed with n-hexane: ethyl acetate at 8:2 (v/v). Fractions (10 ml) were collected and tested by HPLC versus BA standard as described previously [19]. The fraction of highest purity (100 mg) was further characterized by FTIR and liquid chromatography-mass spectrometry (LC-MS).

LC-MS analysis of the BA reference compound, isolated BA, BA-rich fraction and the S.C. methanolic extract was performed using a Micro TOF-Q ESI Mass Spectrometer (Bruker) coupled with a Dionex U3000 HPLC system. Liquid chromatography was carried out on a reverse phase HPLC using Acclaim RSLC C18 column (2.2 μm, 2.1 × 50 mm). The mobile phase consisted of 0.1% formic acid in water (A) and acetonitrile (B), and a gradient elution was used (5–95%) of B in 15 min at a flow rate of 0.3 ml/min. Injection volume was 10 μl, and mass analysis was carried out in the negative ion mode within the range 100–1000 m/z.

#### Quantification of betulinic acid in S.C. methanolic extract

Analytical chromatography was carried out using Agilent 1100 HPLC system, on ZORBAX Eclipse Plus C18 column (5 μm, 4.6 × 250 mm). The mobile phase consisted of A (Acetonitrile), B (0.1% H_3_PO_4_ in water). The elution program was isocratic at 86% (A) and 14% (B) for 20 min, at a flow rate of 1 ml/min. Injection volume was 10 μl, and the wavelength was 210 nm. BA, ursolic acid (UA) and oleanolic acid (OA) reference compounds were also analyzed. Linear regression equation of BA calibration curve (y = 0.3708x + 9.2599, *R*^*2*^ = 1.0) was then applied to calculate BA concentration in the S.C. extract, and the results are presented as %w/w.

### Rat aortic rings angiogenesis model

Antiangiogenesis effect was firstly investigated using the 3-dimensional rat aortic rings model as described previously [15,20,21]. Basically, aortic rings were seeded in 48-well plate containing 500 μl M199 medium containing fibrinogen (3.0 mg/ml), aprotinin (5.0 μg/ml) and L-glutamine (1.0% w/v), followed by addition of 10 μl thrombin (50 U/L). After 90 min incubation, 500 μl M199 medium was added; this medium was supplemented with FBS (20% v/v), L-glutamine (2.0 mM), 6-aminocaproic acid (1.0 mg/ml), amphotericin B (2.5 μg/ml), gentamicin (60 μg/ml), and the treatment compounds. After 4-days incubation at 37°C and 5% CO_2_, the upper layer medium was replaced with a fresh one. On day 5, the distance of outgrowth of the sprouting microvessels was measured [15,22], and the mean percentage growth inhibition was calculated (n = 3).

### Cell viability

Cell viability was determined by the XTT test as described previously [23]. Briefly, cells were treated for 48 h, the old culture medium was replaced with a fresh one containing XTT (100 μg/ml) and PMS (1.0 μg/ml), and incubated for 4 h. Absorbance was then measured at 450 nm using a microplate reader (Thermo Fisher Scientific, Ratastie, Vantaa, Finland). The results are presented as percentage inhibition to the negative control (0.5% DMSO) as the following:

$$\begin{array}{l} \text{Percentage}\;\text{inhibition}\;=(1-\left(OD_{\text{samples}}-OD_{\text{blank}}\right)/\\ \;\left(OD_{\text{Vehicle}}-OD_{\text{blank}}\right))\times 100 \end{array}$$

The median inhibitory concentrations (IC_50s_) were calculated using the dose response curves (n = 3).

### HUVECs tube formation on matrigel matrix

Matrigel tube formation of HUVECs was investigated as previously described with minor modifications [24,25]. In brief, 2 × 10^5^ treated HUVECs (150 μl) were added to 48-well plate containing 150 μl solidified matrigel and incubated for 6 h. Subsequently, the tube-like structures were visualized and photographed under light microscopy at 4× magnification. The images were analyzed by measuring the area occupied by the tube-like structures using ScnImage software package (available free online). The results are presented as percentage inhibition to untreated cells, and the IC_50_ was calculated using the dose response curve (n = 4).

### Determination of VEGF concentration in HUVECs lysates

Concentration of human VEGF-165 in HUVEC cell lysates was determined by human VEGF ELISA kit according to manufacturer’s instructions. HUVECs were treated for 6 h, washed with PBS, and cell lysates were prepared. Calibration curve of VEGF standard was prepared simultaneously, and concentration of VEGF in cell lysates was then determined by applying the VEGF linear regression equation, y = 0.0021x + 0.0585, *R*^*2*^ = 0.999 (n = 3).

### Cell migration

Effect of S.C. extract on HUVECs migration was studied by the wound healing assay as described previously [26]. Briefly, cells were seeded in 6-well plates at 1 × 10^6^ cells/well in 3.0 ml medium, and were allowed to reach 100% confluency. Subsequently, the cell monolayer was scratched with a sterile 200 μl micropipette tip, washed with PBS, and 3.0 ml fresh medium containing treatments was added. The wounds were photographed (8 microscopic fields per well at × 4 magnification using inverted light microscope) immediately and after a specified period of treatment. The distance of cell-free wounds was measured by Leica Quin software in a minimum of 20 points/field, and the percentage of wound closure was calculated relative to zero time treatment using the formula;

$$\begin{array}{l} \%\;\text{Wound}\;\text{closure}\;=(1-(\text{distance}\;\text{at}\;\mathrm{xh}/\\ \;\text{distance}\;\text{at}\;\text{zero}\;\text{time}))\times 100 \end{array}$$

Where *x* refers to the treatment time in hours.

### Chicken embryo chorioallantoic membrane assay

The chicken embryo chorioallantoic membrane (CAM) assay was performed as described previously [27,28]. Fertile eggs were incubated for 5 days at 37°C in a humidified incubator with intermittent manual rotation. On day 5, the large blunt edge was covered with a small piece of adhesive tape, where a small hole was made and 2–5 ml albumin was withdrawn and the eggs were incubated horizontally for 2 h. Subsequently, the eggs were covered with adhesive tape and a circular window was made. Treatments were prepared in ethanol at 20 mg/ml and applied on Whattman filter paper discs at 200 and 100 μg/disc; discs for negative control received the same volume of ethanol. Ethanol was evaporated and the discs were applied directly onto the CAM through the window (n = 12). After 24 h, CAMs were illuminated and photographed under dissecting microscope.

### *In vivo* antitumor effect

Sixteen mice aged 6–8 weeks with average weight of 25 g were injected subcutaneously, in right flank, with 5 × 10^6^ HCT 116 cells in 150 μl RPMI medium. After 7–10 days, animals with uniform tumor size were divided into 2 groups of 5–6 animals. Treatment was performed by mixing S.C. extract with animal food at 0.25% (w/w), and tumor dimensions were measured at 7-days intervals by a caliber in 2 angles, length and width [29]. Tumor size was then calculated as described previously [29-31] using the following equation;

$$\text{Tumor}\;\text{size}\;\left(mm^{3}\right)=\left({\left(\left(W+L\right)/2\right)}^{\wedge }3\right)\times 2$$

Where W is the width and L is the length.

After 28-days treatment, animals were euthanized and the tumors were collected, cross sectioned, and stained with eosin/hematoxylin for microscopic examination.

### Experimental animals

Athymic NCR nu/nu nude mice were obtained from Taconic Farms Inc., USA. Mice were housed in specific pathogen free (SPF) cages, and supplied with autoclaved food, water and bedding. The procedures were approved by USM Animal Ethics Committee (Ref. PPSG/07(A)/044/(2010)(59)).

Sprague Dawley male rats were obtained from animal breeding facility at USM. Animals were euthanized by CO_2_ and the thoracic aortas were collected. Experiments were performed according to the guidelines of USM Animal Ethics Committee and had their approval (Ref. USM/PPSF/50 (084) Jld.2).

### Statistical analysis

The results are presented as mean ± SD. Differences between groups were analyzed by the student t-test or One-way ANOVA and were considered significant at *P* < 0.05.

## Results and discussion

### Phytochemical analysis

The methanolic extract of S.C. was obtained at relatively high percentage yield (16.4%, w/w). Preliminary phytochemical screening showed presence of high concentration of total phenolics (38 ± 1.3%), total flavonoids content (30 ± 3.7%), and also showed a potent DPPH scavenging activity, IC_50_ 33.0 ± 1.0 μg/ml.

### FTIR and UV-Vis spectroscopy fingerprints

S.C. extract was analyzed by FTIR and UV-Vis spectroscopy (Additional file 1: Figures S1 and S2). In FTIR the strong and broad band at 3314 cm^-1^ corresponds to stretching vibration of O–H group, the peak at 2930 cm^-1^ refers to stretching vibration of aliphatic chains, peaks at 1615 cm^-1^, 1521 cm^-1^, and 1449 cm^-1^ correspond to C–C stretching in aromatic rings, the peak at 1697 cm^-1^ corresponds to stretching vibration of the carbonyl group C = O, the bands at 1351 cm^-1^ and 1232 cm^-1^ refer to –C–OH deformation vibrations, and the band at 1044 cm^-1^ refers to –C–OH stretching vibrations. The UV-Vis spectrum showed absorption maxima at 273.1 nm.

### Isolation and characterization of betulinic acid

**Figure 3:**
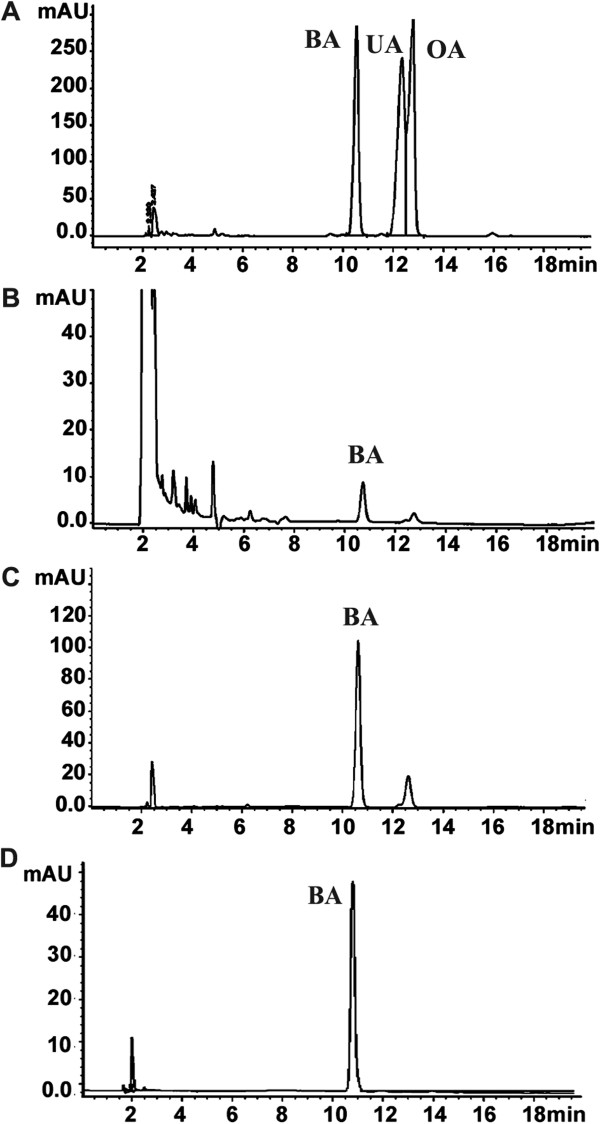
**HPLC analysis of S.C. methanolic extract.** BA, UA and OA standard compounds **(A)**, *S. campanulatum* methanolic extract **(B)**, BA-rich fraction **(C)**, and isolated BA **(D)**.

BA-rich fraction (72% purity) was obtained at relatively high percentage yield (5%) by repeated crystallization and washing of the S.C. methanolic. BA of higher purity (95%) was then isolated from the BA-rich fraction by column chromatography at a percentage yield of 10%. Identity of BA was firstly confirmed by comparing its HPLC retention time (10.6 ± 0.02 min) with BA reference compound (Rt: 10.54 ± 0.02 min). The BA-rich fraction also contained low content of oleanolic acid (Rt: 12.4 ± 0.1 min) and traces of ursolic acid (Rt: 12.7 ± 0.1 min) (Figure 3).

Identity of BA was then confirmed by FTIR analysis; the dominant absorbance bands are located at 3450, 2939, 2872, 1689, 1642, 1455, 1377, 1232, 1190, 1142, 1036, and 884 cm^-1^. By comparing the band positions of BA standard with isolated BA, identical spectra were obtained which confirm the identity of isolated BA (Additional file 1: Figure S3). BA identity was further confirmed by MS analysis; BA reference and isolated compound was eluted at the same retention time (10.56 min), and the mass spectral isotopic pattern of isolated BA matches that of reference BA (455.35, 456.35 and 457,35 m/z). LC-MS analysis of methanolic extract also showed presence of compound with a mass of 455.35 m/z (Additional file 1: Figure S5). Taken together these results confirm presence of BA in S.C. methanolic leaf extract. HPLC quantitative analysis of BA in the S.C. extract indicates presence of the compound at 5.42 ± 0.09% (w/w).

### *In vitro* antiangiogenesis effect

**Figure 4:**
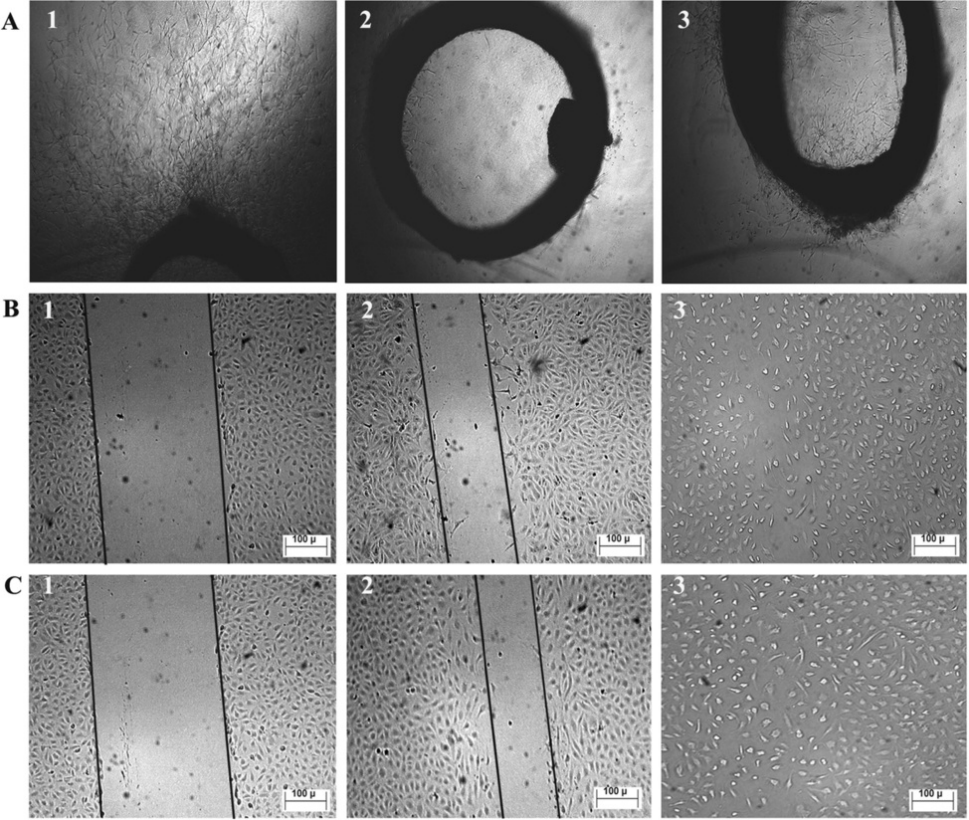
***In vitro *inhibition of angiogenesis.** Rat aortic rings **(A)**: Untreated (1), suramin at 100 μg/ml (2), and S.C. methanolic extract at 100 μg/ml (3). Inhibition of HUVECs cell migration **(B** and **C)**: zero time treatment (**B**1 and **C**1), methanolic extract at 100 μg/ml (**B**2) and at 50 μg/ml (**C**2), Untreated cells (**B**3 and **C**3). The lines are drawn for explanation.

Antiangiogenesis effect of S.C. extract was studied by various tests that target different angiogenesis hallmarks. Preliminary testing was performed on rat aortic rings which involve all steps of the angiogenesis cascade except blood flow. The results showed strong inhibition of microvessels outgrowth at 100 μg/ml (65 ± 11)%, compared to 0.0 ± 10.7% by the vehicle (0.5% DMSO) and 100 ± 1.0% by suramin at 100 μg/ml (Figure 4A). However, this inhibitory effect can be due to nonselective cytotoxic or interference with the angiogenesis cascade. In order to make distinction between both possibilities, cytotoxicity of S.C. extract was evaluated on endothelial versus other normal and cancer cells. The extract, at the same concentration used in rat aortic rings test (100 μg/ml), did not show any significant cytotoxicity on all tested cell lines; the percentage of growth inhibition was 0.0 ± 4.0% (HUVECs), 2.0 ± 1.0% (MCF-10A), 2.0 ± 3.0% (CCD-18Co), 12 ± 0.0% (MCF-7), and 15 ± 2.0% (HCT 116). These results indicate the extract is noncytotoxic; hence we can be conclude that inhibition of microvessels outgrowth observed in rat aortic rings is not due to nonselective cytotoxicity, but due to interference with angiogenesis process.

**Figure 5:**
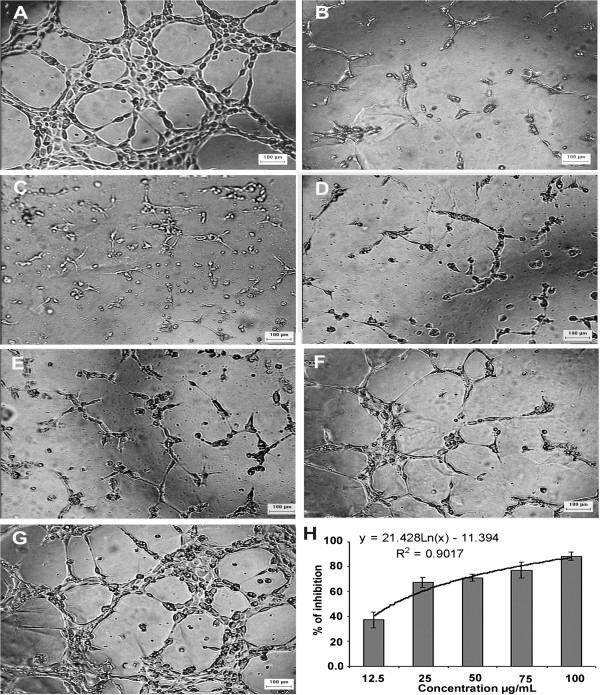
**Inhibition of tube formation on Matrigel matrix.** Untreated cells **(A)**, suramin **(B)**, **(C-G)** S.C. methanolic extract at 100, 75, 50, 25 and 12.5 μg/ml, and analysis showing a dose dependent inhibition of tube formation **(H)**.

Antiangiogenic effect was further studied using HUVECs tube formation on matrigel matrix which measures differentiation of endothelial cells. Matrigel is a tumor-derived matrix that contains all components present in basement membranes and the growth factors required to promote differentiation of endothelial cells to start forming blood vessels-like structures [32,33]. Our results showed potent inhibition of tube formation in dose dependent manner with IC_50_ of 17.6 ± 2.9 μg/ml (Figure 5). Inhibition of tube formation may be mediated by interference with plasminogen activators (PAs), matrix metalloproteinases MMPs, growth factors, and cell adhesion molecules [34,35].

VEGF is a key growth factor overexpressed in most solid tumors; it initiates angiogenesis process that is required for tumor growth and metastasis by inducing proliferation, migration, sprouting and tube formation of endothelial cells [36-39]. Therefore, suppression of VEGF expression is considered as a good target in treatment of angiogenesis dependent diseases. In this study effect on VEGF expression in endothelial cells was investigated as a possible mechanism of S.C. extract antiangiogenic effect; the results showed significant suppression of VEGF expression in HUVECs lysates at 100 μg/ml (12 ± 1.8 pg/ml) compared to untreated cells (26 ± 2.7 pg/ml), *P* = 0.001.

Cell migration study showed significant and potent inhibition of HUVECs migration by S.C. extract at 100 and 50 μg/ml (Figure 4B and C). It is noteworthy that S.C. extract was more potent than suramin, a standard angiogenesis inhibitor (Table 1), *P* = 0.001.

**Table 1:** S.C. methanolic extract Inhibits HUVECS migration

**Time (h)**	**% Wound Closure**			
	**0.5% DMSO**	**100 μg/mLl**	**50 μg/ml**	**Suramin 25 μg/ml**
12	74 ± 10%	36 ± 6%	50 ± 7%	53 ± 5%
18	87 ± 6%	41 ± 4%	64 ± 7%	84 ± 6%

Results are presented as percentage of wound closure ± SD (n = 3).

### *In vivo* antiangiogenesis effect

**Figure 6:**
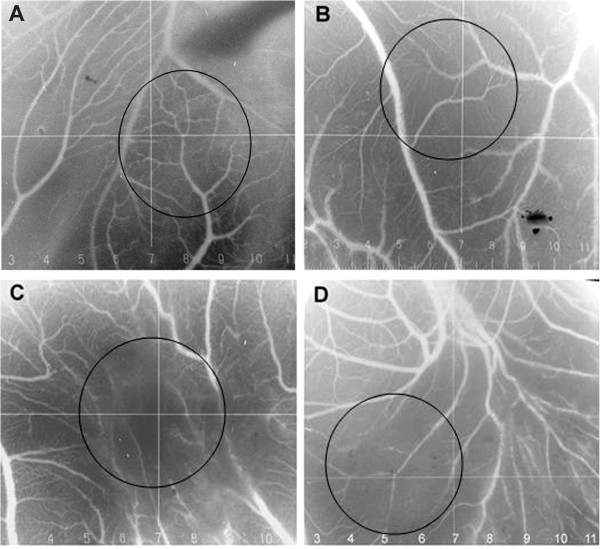
***In vivo *inhibition of angiogenesis using Chicken embryo CAMs.** Untreated CAM **(A)**, treated with suramin at 25 μg/disc **(B)**, treated with S.C. methanolic extract at 200 μg/disc **(C)** and 100 μg/disc **(D)**.

*In vivo* antiangiogenic effect was investigated on chicken embryo CAMs. Results show inhibition of CAMs vascularization by S.C. extract at 200 and 100 μg/disc. Figure 6A shows normal vascularization of untreated CAMs with primary, secondary and tertiary vessels and dendritic branching. On the other hand, CAMs treated with S.C. extract showed distorted vasculature or absence of blood vessels (Figure 6B-D).

Taken together, inhibition of microvessels outgrowth in rat aortic rings, inhibition of HUVECs migration, inhibition of tube formation on matrigel matrix and interference with vascularization of chicken embryo CAMs provide evidence about antiangiogenic effect of S.C. extract, which can be explained due to suppression of VEGF expression in endothelial cells.

Phytochemical analysis of S.C. extract showed presence of high phenolics, flavonoids and BA content, which may explain the antiangiogenic effect of S.C. extract. Antiangiogenic effect of phenolic compounds such as flavonoids has been reported by several research groups worldwide, with different mechanisms of action such as suppression of VEGF and HIF-1α expression [40]. Recent studies that explored the mechanism of BA antiangiogenic effect in different types of cancer concluded that the compound inhibits tumor angiogenesis by suppressing expression of the signal transducer and activator of transcription 3 (STAT3), hypoxia inducible factor-1α and VEGF [41,42].

### *In vivo* antitumor effect

**Figure 7:**
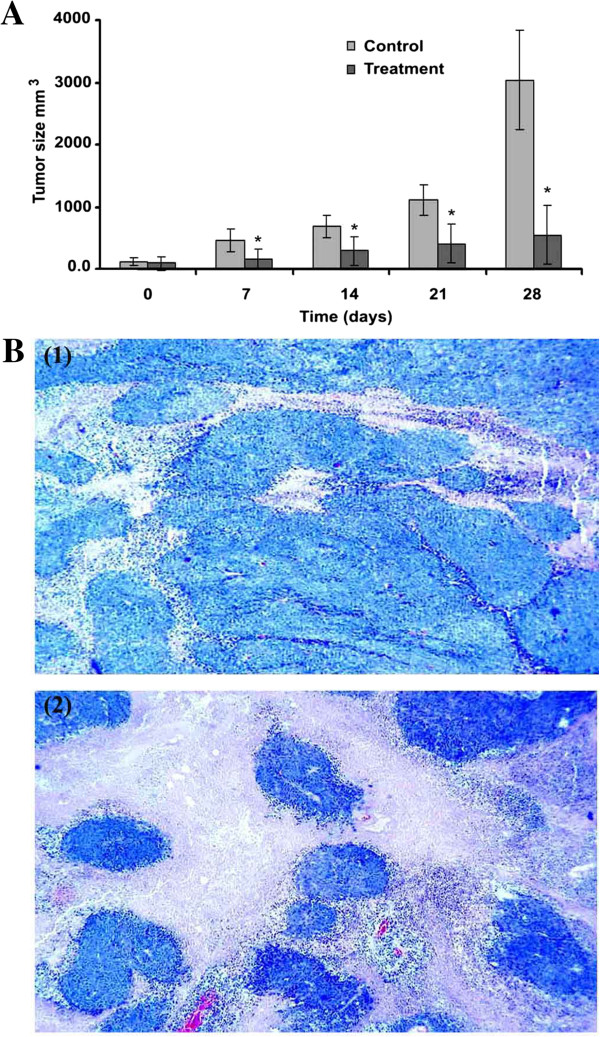
**Inhibition of the subcutaneous tumor growth in nude mice.** A time course of tumor size measurement **(A)**, cross sections of tumor tissues **(B)**: untreated (1), and treated with S.C. methanolic extract at 0.25% w/w (2). Pictures were taken at 5× magnification. (*) refers to *P* < 0.05.

HCT 116 cells provides an invasive model of human colorectal carcinoma [43], which depends highly on angiogenesis for the tumor to grow and metastasize [44]. *In vivo* antitumor study showed significant inhibition of tumor growth by treatment with S.C. methanolic extract at 0.25 w/w% (Figure 7A). Microscopic examination of tumor cross sections showed significant reduction in the number of intratumor blood vessels in treated (4.6 ± 0.5/microscopic field) compared to untreated animals (7.8 ± 1.2/microscopic filed), *P* = 0.001. Moreover, histological examination of tumor cross sections revealed more abundant apoptotic/necrotic regions in tumors of treated animals compared to untreated animals (Figure 7B). It is noteworthy that S.C. extract did not show obvious toxicity to treated mice, and no significant difference was observed in weight gain between treated (0.3 ± 7.0)% and untreated animals (5.0 ± 4.0)%, *P* = 0.001. Anti-colon cancer effect of S.C. extract may be attributed to inhibition of tumor angiogenesis which often leads to decreased nutrient and oxygen supply, and consequently decreased tumor growth, increased extent of tumor necrosis and ultimately decreased tumor size.

## Conclusion

Collectively, our data showed that S.C. methanolic extract is rich in phenolics, flavonoids and betulinic acid. The extract suppressed expression of VEGF in endothelial cells, and inhibited angiogenesis and tumor growth in nude mice. A possible mechanism of the anti-colon cancer activity of S.C. extract is the inhibition of tumor angiogenesis. Antiangiogenic and antitumor effects of S.C. can be explained, at least partly, due to the high flavonoids and betulinic acid content. Thus, our findings suggest that S.C. extract could be an interesting antiangiogenic candidate that targets the VEGF signaling pathway. *S. campanulatum* may have applications in colon cancer adjuvant therapy and other angiogenesis related diseases.

## Competing interests

The authors declared that they have no competing interests.

## Authors’ contributions

AFA designed, carried out the experiments, performed the statistical analysis, and prepared the manuscript. KM helped in editing the manuscript. ZI helped in phytochemical analysis. GG helped in HPLC analysis. MJS helped in LC-MS analysis. AMS developed the concept and edited the manuscript. All authors read and approved the final manuscript.

## Pre-publication history

The pre-publication history for this paper can be accessed here:

http://www.biomedcentral.com/1472-6882/13/168/prepub

## Supplementary Material

Additional file 1: Figure S1 FT-IR spectrum of *S. campanulatum* methanolic extract. **Figure S2** UV-VIS spectrum of *S. campanulatum* methanolic extract. **Figure S3** An overlay of FTIR spectra of isolated and standard betulinic acid. **Figure 4S** LC-MS analysis of betulinic acid. Standard compound from Sigma (A), isolated compound from *S. campanulatum* methanolic extract (B), BA-rich fraction from *S. campanulatum* methanolic extract (C), isotopic pattern of standard betulinic acid (D) and isolated betulinic acid (F). **Figure 5S** LC-MS analysis of betulinic acid in *S. campanulatum* methanolic extract. Betulinic acid is compound number 18 with a retention time of 10.57 min, a mass spectral isotopic pattern of (M-1) 455.3532, 456.3561, and 457.3594 m/z, and molecular formula C_30_H_48_O_3_. Click here for file

## References

[B1] Roseli ANM Ying TF Ramlan MF Growth inhibition of syzygium campanulatum korth. For container planting by the application of uniconazole Pertanika J Trop Agric Sci 2010 33 1 6

[B2] Folkman J Angiogenesis in cancer, vascular, rheumatoid and other disease Nat Med 1995 1 27 31 10.1038/nm0195-27 7584949

[B3] Pepper MS Manipulating angiogenesis: from basic science to the bedside Arterioscler Thromb Vasc Biol 1997 17 605 10.1161/01.ATV.17.4.605 9108772

[B4] Folkman J Tumor angiogenesis: therapeutic implications N Engl J Med 1971 285 1182 1186 10.1056/NEJM197111182852108 4938153

[B5] Kanjoormana M Kuttan G Antiangiogenic activity of ursolic acid Integr Cancer Ther 2010 9 224 235 20462855 10.1177/1534735410367647

[B6] Sogno I Vannini N Lorusso G Cammarota R Noonan DM Generoso L Sporn MB Albini A Anti-angiogenic activity of a novel class of chemopreventive compounds: oleanic acid terpenoids Recent Results Cancer Res 2009 181 209 212 10.1007/978-3-540-69297-3_19 19213570

[B7] You YJ Nam NH Kim Y Bae KH Ahn BZ Antiangiogenic activity of lupeol from Bombax ceiba Phytother Res 2003 17 341 344 10.1002/ptr.1140 12722136

[B8] Mukherjee R Jaggi M Rajendran P Siddiqui MJA Srivastava SK Vardhan A Burman AC Betulinic acid and its derivatives as anti-angiogenic agents Bioorg Med Chem Lett 2004 14 2181 2184 10.1016/j.bmcl.2004.02.044 15081004

[B9] Leong H Mathur PS Greene GL Green tea catechins inhibit angiogenesis through suppression of STAT3 activation Breast Cancer Res Treat 2009 117 505 515 10.1007/s10549-008-0196-x 18821062 PMC3664280

[B10] Cao Y Fu ZD Wang F Liu HY Han R Anti-angiogenic activity of resveratrol, a natural compound from medicinal plants J Asian Nat Prod Res 2005 7 205 213 10.1080/10286020410001690190 15621628

[B11] Tan WF Lin LP Li MH Zhang YX Tong YG Xiao D Ding J Quercetin, a dietary-derived flavonoid, possesses antiangiogenic potential Eur J Pharmacol 2003 459 255 262 10.1016/S0014-2999(02)02848-0 12524154

[B12] Huang SS Zheng RL Rosmarinic acid inhibits angiogenesis and its mechanism of action in vitro Cancer Lett 2006 239 271 280 10.1016/j.canlet.2005.08.025 16239062

[B13] Su SJ Yeh TM Chuang WJ Ho CL Chang KL Cheng HL Liu HS Hsu PY Chow NH The novel targets for anti-angiogenesis of genistein on human cancer cells Biochem Pharmacol 2005 69 307 318 10.1016/j.bcp.2004.09.025 15627483

[B14] Arbiser JL Klauber N Rohan R van Leeuwen R Huang MT Fisher C Flynn E Byers HR Curcumin is an in vivo inhibitor of angiogenesis Mol Med 1998 4 376 383 10780880 PMC2230271

[B15] Aisha AFA Abu-Salah KM Darwis Y Abdul Majid AMS Screening of antiangiogenic activity of some tropical plants by rat aorta ring assay Int J Pharamcol 2009 5 370 376

[B16] Lizcano LJ Bakkali F Begoña Ruiz-Larrea M Ignacio Ruiz-Sanz J Antioxidant activity and polyphenol content of aqueous extracts from Colombian Amazonian plants with medicinal use Food Chem 2010 119 1566 1570 10.1016/j.foodchem.2009.09.043

[B17] Kosalec I Bakmaz M Pepeljnjak S Vladimir-Knezevic S Quantitative analysis of the flavonoids in raw propolis from northern Croatia Acta Pharmaceutica-Zagreb 2004 54 65 72 15050046

[B18] Sharma OP Bhat TK DPPH antioxidant assay revisited Food Chem 2009 113 1202 1205 10.1016/j.foodchem.2008.08.008

[B19] Aisha AFA Abu-Salah KM Alrokayan SA Siddiqui MJ Ismail Z Abdul Majid AMS Syzygium aromaticum extracts as good source of betulinic acid and potential anti-breast cancer Rev Bras Farmacogn 2012 22 335 343 10.1590/S0102-695X2011005000185

[B20] Brown KJ Maynes SF Bezos A Maguire DJ Ford MD Parish CR A novel in vitro assay for human angiogenesis Lab Invest 1996 75 539 555 8874385

[B21] Aisha AFA Nassar ZD Siddiqui MJ Abu-Salah KM Alrokayan SA Ismail Z Abdul Majid AMS Evaluation of antiangiogenic, cytotoxic and antioxidant effects of Syzygium aromaticum L. extracts Asian J Biol Sci 2011 4 282 290

[B22] Nicosia RF Lin YJ Hazelton D Qian X Endogenous regulation of angiogenesis in the rat aorta model. Role of vascular endothelial growth factor Am J Pathol 1997 151 1379 1386 9358764 PMC1858079

[B23] Jost LM Kirkwood JM Whiteside TL Improved short- and long-term XTT-based colorimetric cellular cytotoxicity assay for melanoma and other tumor cells J Immunol Methods 1992 147 153 165 10.1016/S0022-1759(12)80003-2 1548398

[B24] Arnaoutova I Kleinman HK In vitro angiogenesis: endothelial cell tube formation on gelled basement membrane extract Nat Protoc 2010 5 628 635 20224563 10.1038/nprot.2010.6

[B25] Aisha AF Abu-Salah KM Alrokayan SA Ismail Z Abdulmajid AM Evaluation of antiangiogenic and antoxidant properties of Parkia speciosa Hassk extracts Pak J Pharm Sci 2012 25 7 14 22186303

[B26] Liang CC Park AY Guan JL In vitro scratch assay: a convenient and inexpensive method for analysis of cell migration in vitro Nat Protoc 2007 2 329 333 17406593 10.1038/nprot.2007.30

[B27] Dohle DS Pasa SD Gustmann S Laub M Wissler JH Jennissen HP Nker N Chick ex ovo culture and ex ovo CAM assay: How it really works J Vis Exp 2009 33 1620 19949373 10.3791/1620 PMC3157849

[B28] Nassar ZD Aisha AF Ahamed MB Ismail Z Abu-Salah KM Alrokayan SA Abdul Majid AM Antiangiogenic properties of Koetjapic acid, a natural triterpene isolated from Sandoricum koetjape Merr Cancer Cell Int 2011 11 12 10.1186/1475-2867-11-12 21524294 PMC3111336

[B29] Tomayko MM Reynolds CP Determination of subcutaneous tumor size in athymic (nude) mice Cancer Chemother Pharmacol 1989 24 148 154 10.1007/BF00300234 2544306

[B30] Kopper L Steel GG The therapeutic response of three human tumor lines maintained in immune-suppressed mice Cancer Res 1975 35 2704 2713 1157045

[B31] Fodstad O Aamdal S Pihl A Boyd MR Activity of mitozolomide (NSC 353451), a new imidazotetrazine, against xenografts from human melanomas, sarcomas, and lung and colon carcinomas Cancer Res 1985 45 1778 1786 3978640

[B32] Kleinman HK Martin GR Matrigel: basement membrane matrix with biological activity Semin Cancer Biol 2005 15 378 386 10.1016/j.semcancer.2005.05.004 15975825

[B33] Benton G Kleinman HK George J Arnaoutova I Multiple uses of basement membrane-like matrix (BME/Matrigel) in vitro and in vivo with cancer cells Int J Cancer 2011 128 1751 1757 10.1002/ijc.25781 21344372

[B34] Mignatti P Rifkin DB Plasminogen activators and matrix metalloproteinases in angiogenesis Enzyme Protein 1996 49 117 137 8797002 10.1159/000468621

[B35] Bischoff J Cell adhesion and angiogenesis J Clin Invest 1997 99 373 376 10.1172/JCI119168 9022067 PMC507807

[B36] Ferrara N Gerber HP LeCouter J The biology of VEGF and its receptors Nat Med 2003 9 669 676 10.1038/nm0603-669 12778165

[B37] Lamalice L Le Boeuf F Huot J Endothelial cell migration during angiogenesis Circ Res 2007 100 782 794 10.1161/01.RES.0000259593.07661.1e 17395884

[B38] Nishi M Abe Y Tomii Y Tsukamoto H Kijima H Yamazaki H Ohnishi Y Iwasaki M Inoue H Ueyama Y Nakamura M Cell binding isoforms of vascular endothelial growth factor-A (VEGF189) contribute to blood flow-distant metastasis of pulmonary adenocarcinoma Int J Oncol 2005 26 1517 1524 15870864 10.3892/ijo.26.6.1517

[B39] Brogi E Schatteman G Wu T Kim EA Varticovski L Keyt B Isner JM Hypoxia-induced paracrine regulation of vascular endothelial growth factor receptor expression J Clin Invest 1996 97 469 476 10.1172/JCI118437 8567969 PMC507039

[B40] Mojzis J Varinska L Mojzisova G Kostova I Mirossay L Antiangiogenic effects of flavonoids and chalcones Pharmacol Res 2008 57 259 265 10.1016/j.phrs.2008.02.005 18387817

[B41] Shin J Lee HJ Jung DB Jung JH Lee EO Lee SG Shim BS Choi SH Ko SG Suppression of STAT3 and HIF-1 alpha mediates anti-angiogenic activity of betulinic acid in hypoxic PC-3 prostate cancer cells PLoS One 2011 6 e21492 10.1371/journal.pone.0021492 21731766 PMC3123343

[B42] Karna E Szoka L Palka JA Betulinic acid inhibits the expression of hypoxia-inducible factor 1alpha and vascular endothelial growth factor in human endometrial adenocarcinoma cells Mol Cell Biochem 2010 340 15 20 10.1007/s11010-010-0395-8 20174965

[B43] Rajput A Dominguez San Martin I Rose R Beko A Levea C Sharratt E Mazurchuk R Hoffman RM Brattain MG Wang J Characterization of HCT116 human colon cancer cells in an orthotopic model J Surg Res 2008 147 276 281 10.1016/j.jss.2007.04.021 17961596

[B44] Allen J Bergsland EK Angiogenesis in colorectal cancer: therapeutic implications and future directions Hematol Oncol Clin North Am 2004 18 1087 1119 10.1016/j.hoc.2004.05.002 15474337

