# Supplementary material for: Syzygium campanulatum korth methanolic extract inhibits angiogenesis and tumor growth in nude mice
Source: BMC Complement Altern Med. 2013 Jul 11;13:168. doi: 10.1186/1472-6882-13-168 (PMC3717079; doi:10.1186/1472-6882-13-168)
Supplement: Additional file 1: Figure S1 — FT-IR spectrum of S. campanulatum methanolic extract. Figure S2 UV-VIS spectrum of S. campanulatum methanolic extract. Figure S3 An overlay of FTIR spectra of isolated and standard betulinic acid. Figure 4S LC-MS analysis of betulinic acid. Standard compound from Sigma (A), isolated compound from S. campanulatum methanolic extract (B), BA-rich fraction from S. campanulatum methanolic extract (C), isotopic pattern of standard betulinic acid (D) and isolated betulinic acid (F). Figure 5S LC-MS analysis of betulinic acid in S. campanulatum methanolic extract. Betulinic acid is compound number 18 with a retention time of 10.57 min, a mass spectral isotopic pattern of (M-1) 455.3532, 456.3561, and 457.3594 m/z, and molecular formula C30H48O3. [file 1472-6882-13-168-S1.doc]

***Syzygium campanulatum Korth Methanolic Extract Inhibits Angiogenesis and Tumor Growth in Nude Mice***

Abdalrahim F.A. Aisha^1,2§^, Zhari Ismail^1^, Khalid M. Abu-Salah^3^, Jamshed M. Siddiqui^4^, Gheniya Ghafar^1^, Amin Malik Shah Abdul Majid^2,5§^

^1^Department of Pharmaceutical Chemistry, School of Pharmaceutical Sciences, Universiti Sains Malaysia, Minden 11800, Pulau Pinang, Malaysia

^2^Department of Pharmacology, School of Pharmaceutical Sciences, Universiti Sains Malaysia, Minden 11800, Pulau Pinang, Malaysia

^3^The chair of Cancer Targeting and Treatment, King Abdullah Institute for Nanotechnology, King Saud University, Riyadh 11451, Saudi Arabia

^4^Department of Pharmaceutical Chemistry, International Islamic University Malaysia, Bandar Indera Mahkota Campus, 25200 Kuantan, Pahang, Malaysia

^5^Australian Institute for Nanotechnology and Bioengineering, University of Queensland, Queensland 4072, Australia

^§^Corresponding author

Email addresses

AFAA: [abedaisheh@yahoo.com](mailto:abedaisheh@yahoo.com)

ZI: [zhari@usm.my](mailto:zhari@usm.my)

KMAS: [k_abusalah@hotmail.com](mailto:k_abusalah@hotmail.com)

GG: [gheniyaghafar@yahoo.com](mailto:gheniyaghafar@yahoo.com)

MJS: siddiquijamshed@hotmail.com

AMSAM: [aminmalikshah@gmail.com](mailto:aminmalikshah@gmail.com)


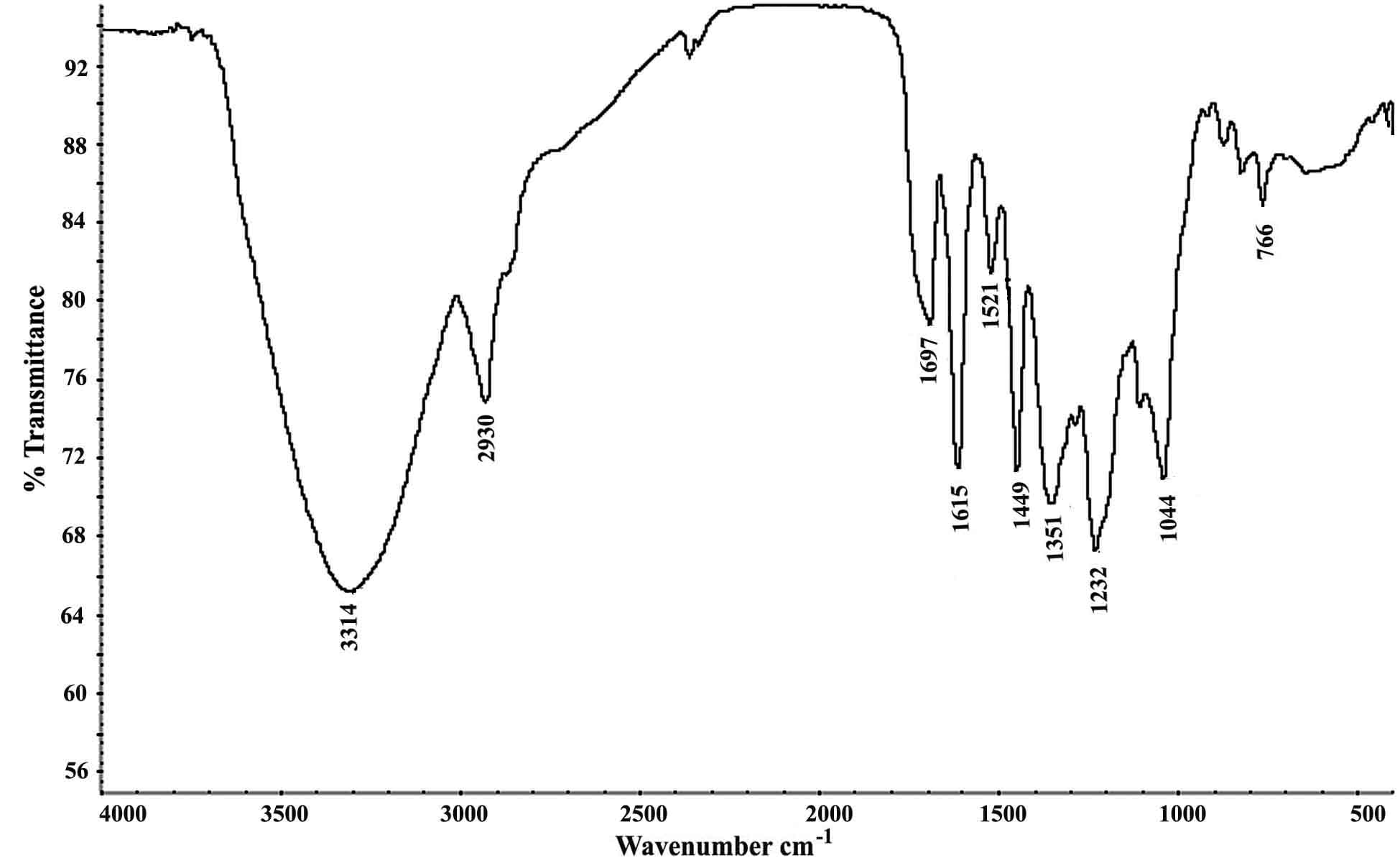


**Additional file 1: Figure S1** FT-IR spectrum of *S. campanulatum* methanolic extract.


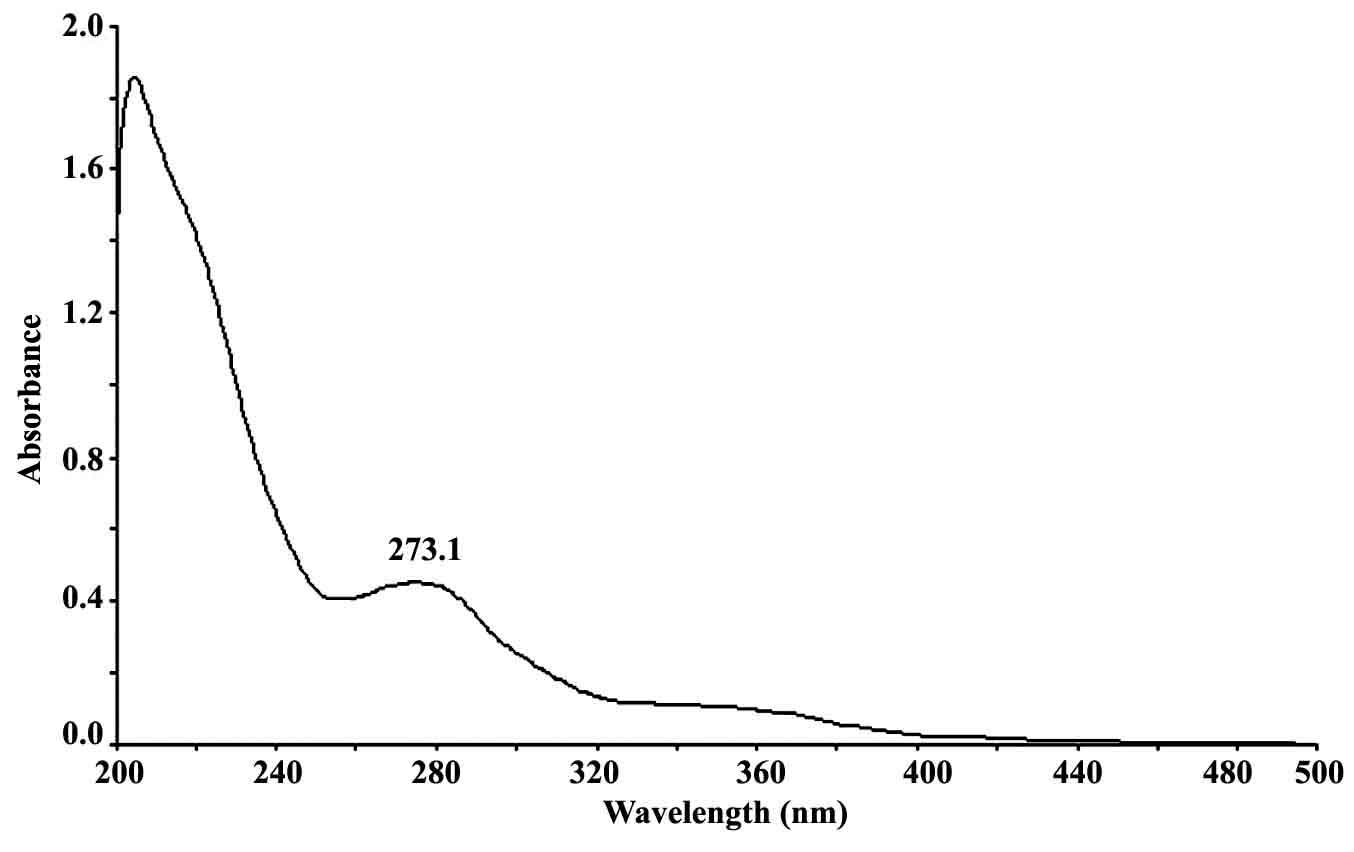


**Additional file 1: Figure S2** UV-VIS spectrum of *S. campanulatum* methanolic extract.


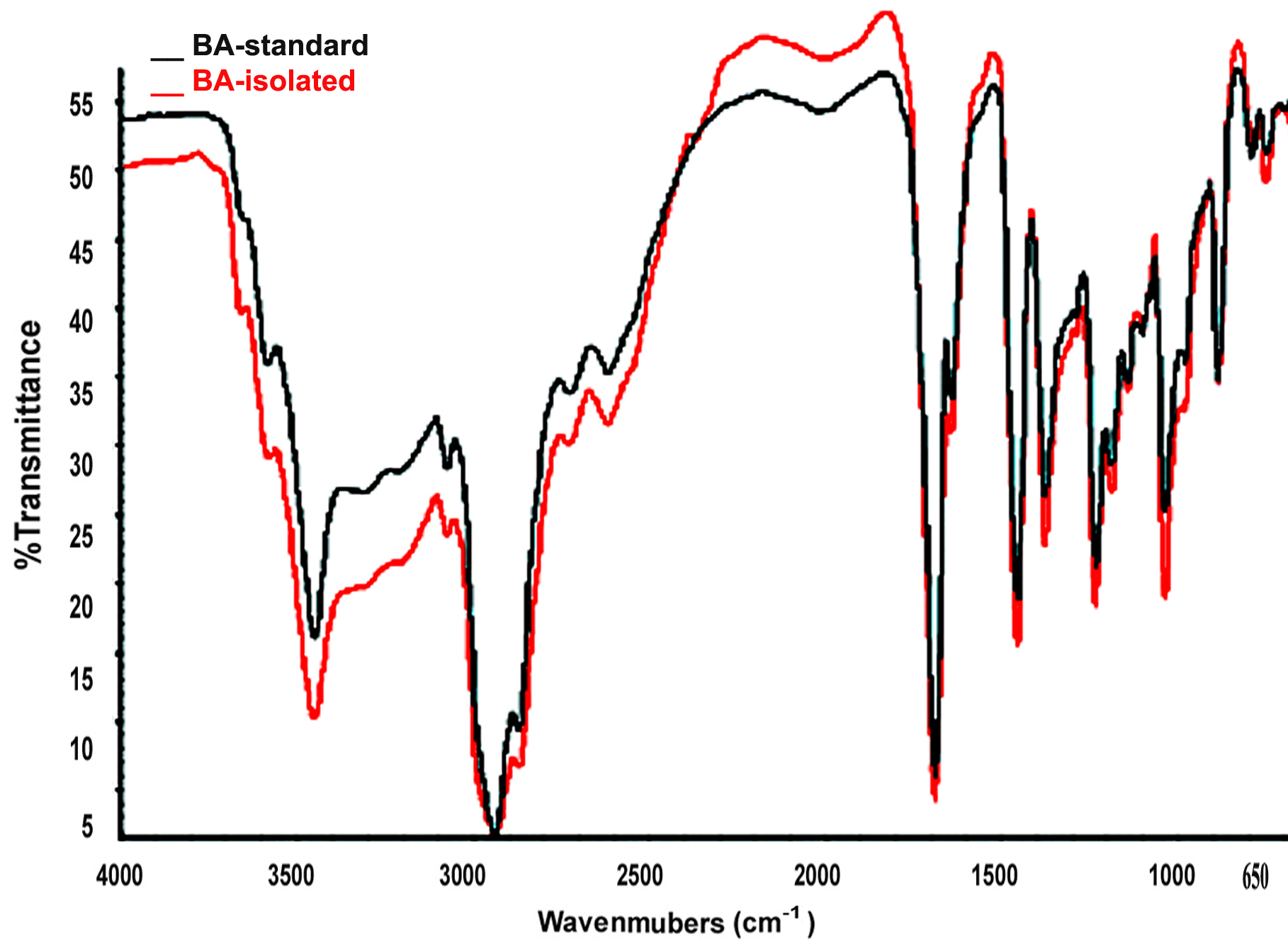


**Additional file 1: Figure S3** An overlay of FTIR spectra of isolated and standard betulinic acid.


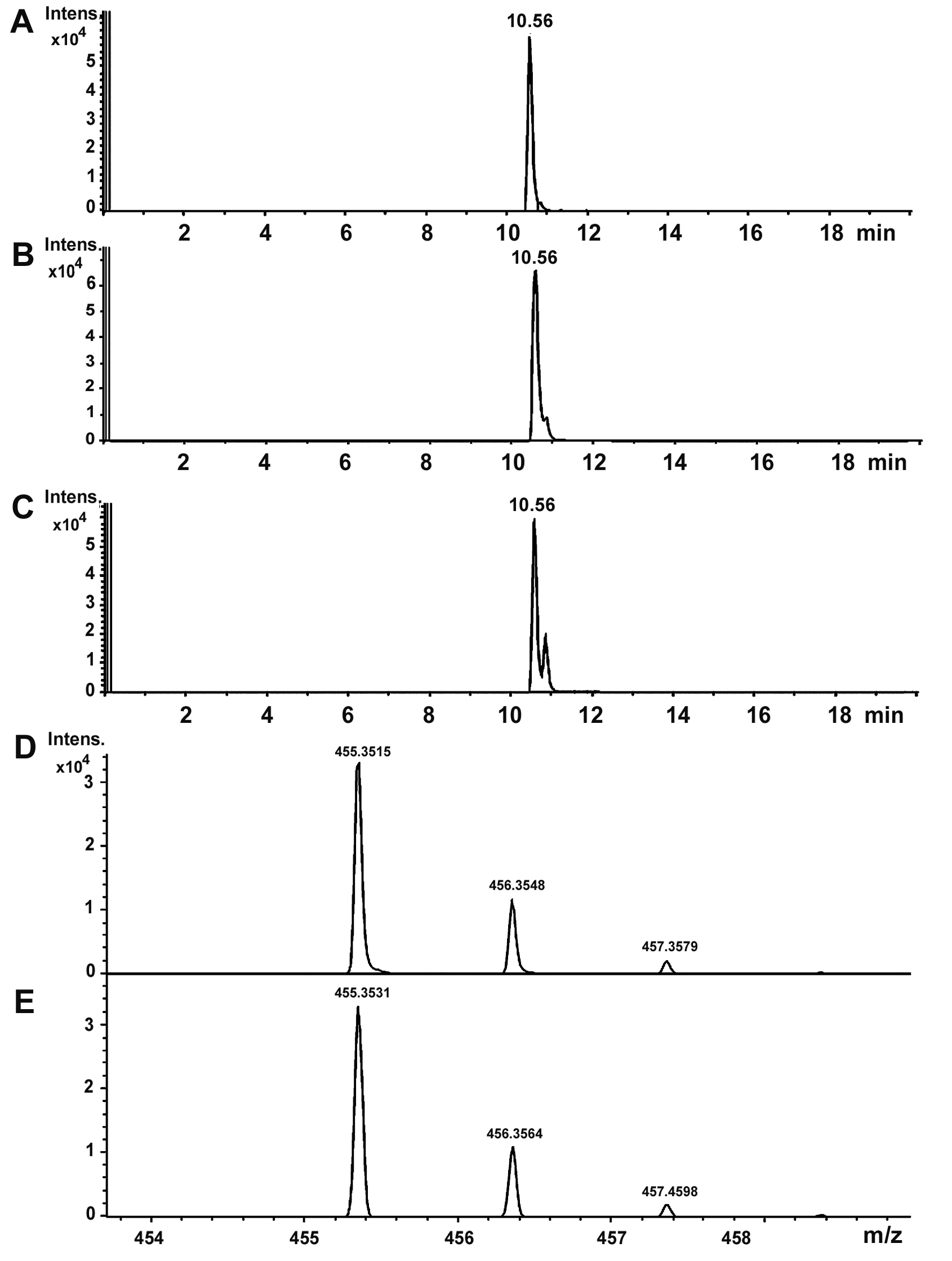


**Additional file 1: Figure S4** LC-MS analysis of betulinic acid. Standard compound from Sigma (A), isolated compound from *S. campanulatum* methanolic extract (B), BA-rich fraction from *S. campanulatum* methanolic extract (C), isotopic pattern of standard betulinic acid (D) and isolated betulinic acid (F).


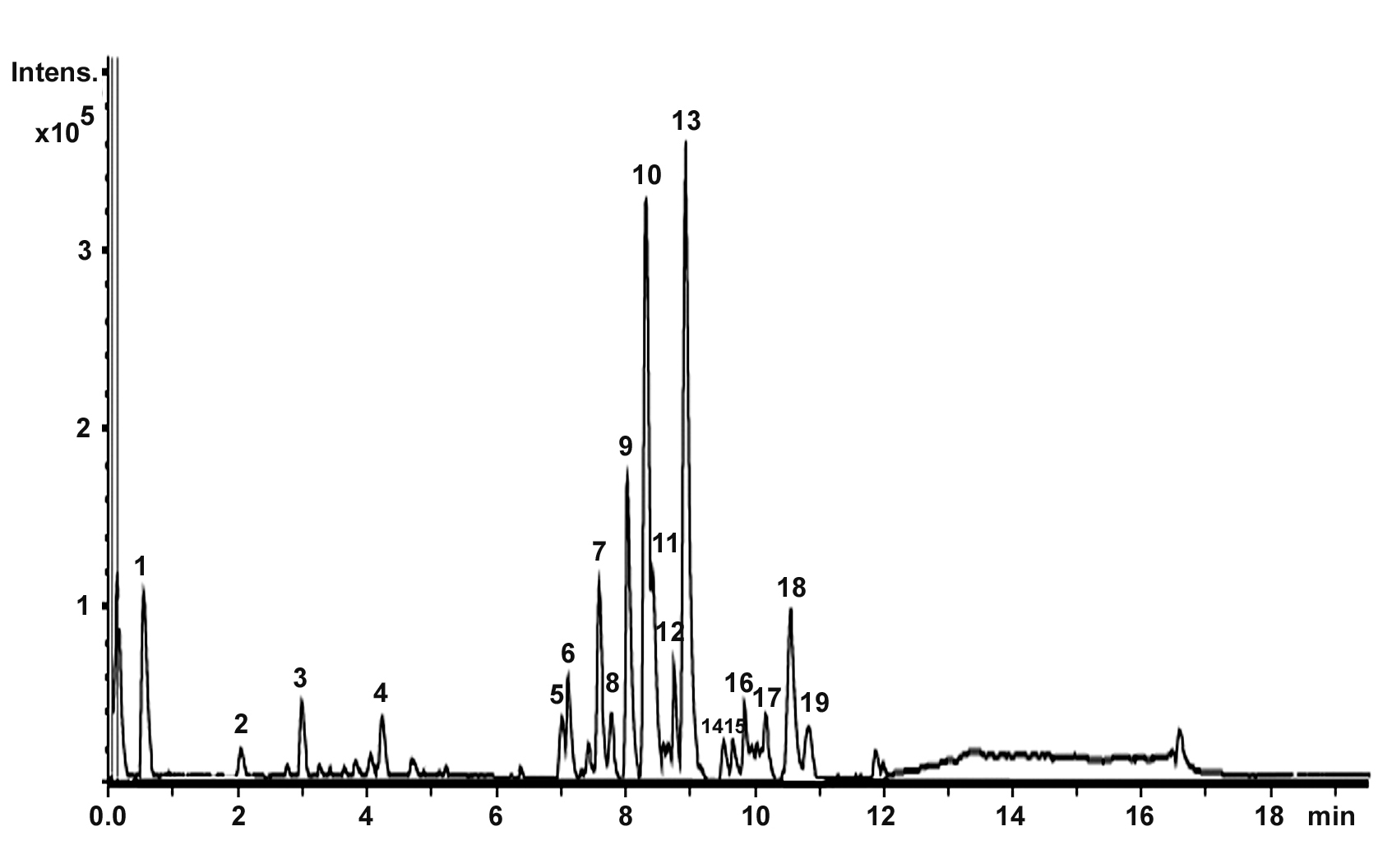


**Additional file 1: Figure S5** LC-MS analysis of betulinic acid in *S. campanulatum* methanolic extract. Betulinic acid is compound number 18 with a retention time of 10.57 min, a mass spectral isotopic pattern of (M-1) 455.3532, 456.3561, and 457.3594 m/z, and molecular formula C_30_H_48_O_3_.
